# Relationship between Selenium and Hematological Markers in Young Adults with Normal Weight or Overweight/Obesity

**DOI:** 10.3390/antiox8100463

**Published:** 2019-10-08

**Authors:** Doreen Yvonne Larvie, Jeanne Lynn Doherty, George L. Donati, Seth Mensah Armah

**Affiliations:** 1 Department of Nutrition, University of North Carolina at Greensboro, Greensboro, NC 27412, USA; dylarvie@uncg.edu (D.Y.L.); jldohert@uncg.edu (J.L.D.); 2 Department of Chemistry, Wake Forest University, Winston-Salem, NC 27109, USA; donatigl@wfu.edu

**Keywords:** anemia of inflammation, hepcidin, iron status, selenoprotein P, glutathione peroxidase, inflammation

## Abstract

Selenium deficiency has been linked to anemia of inflammation, which is mediated by hepcidin. However, there are few studies providing evidence of the role of hepcidin in this relationship. In this study, we investigated the interrelationships among selenium biomarkers, hepcidin concentration, and iron status among individuals with overweight/obesity compared to their normal weight counterparts, since obesity is associated with chronic inflammation. A total of 59 college students were recruited for this study. Fasting blood samples were collected for the analysis of iron status, plasma selenoproteins (glutathione peroxidase (GPX) activity and selenoprotein P (SEPP1)), and plasma hepcidin. Subjects completed three-day dietary records to determine average daily nutrient intakes. SEPP1 concentration, GPX activity, and iron status biomarkers (serum iron, transferrin saturation, and hemoglobin concentration) were lower among individuals with overweight/obesity compared with individuals with normal weight, but these differences were not significant (*p* > 0.05). Regression analysis showed that GPX activity (β = −0.018, *p* = 0.008) and SEPP1 concentration (β = −1.24, *p* = 0.03) were inversely associated with hepcidin concentration. The inverse association between selenoproteins and hepcidin concentration supports a potential role of hepcidin as a mediator between selenium and iron status and warrants further studies to better understand this relationship.

## 1. Introduction

Anemia affects a third of the world’s population [1]. In the US, the prevalence increased by 3% from years 2003 to 2012 [2]. Anemia results from a homeostatic iron imbalance with increased destruction and/or impaired synthesis of erythrocytes [1]. Iron deficiency is the most common cause of anemia accounting for about half of all cases, followed by inflammation [3,4]. 

Inflammation refers to a biological response of the immune system to infection and is a secondary component of chronic diseases [5,6]. Inflammation accounts for about a fifth of anemia cases in older adults in the US and, among individuals with obesity, the observed chronic low-grade inflammation is implicated to result in hypoferremia [1,3,4,7]. Anemia caused by inflammation is associated with alterations in iron metabolism, erythrocyte life span and production, lowered transferrin saturation and serum iron, and increased ferritin concentrations [8]. The link between inflammation and anemia has been explained by the iron regulatory protein, hepcidin. Hepcidin is a 25-amino-acid (disulfide-rich) peptide that plays a critical role in iron metabolism by acting as a signaling molecule, and in immunity due to its antimicrobial properties [9,10]. Hepcidin acts by binding to the iron transporter (ferroportin) resulting in its degradation [11]. Hepcidin concentration increases in response to high iron stores and inflammation and decreases with anemia and hypoxia [12]. In obesity, macrophages and immune system cells invade the adipose tissue in response to fat accumulation leading to the production of proinflammatory cytokines including interleukin 6 (IL-6) [13]. With the increased production of IL-6, hepcidin is upregulated leading to a reduction in circulating iron, an increase in iron storage in macrophages and hepatocytes, and a consequent mild iron deficiency [14,15]. 

Due to oxidative stress and inflammation in obesity, there is increased need for antioxidant nutrients such as selenium [16,17]. Selenium is incorporated as part of selenocysteine at the active site of selenoproteins (glutathione peroxidase (GPX), selenoprotein P (SEPP1), and thioredoxin reductase). GPX refers to a family of antioxidant enzymes that protects cells against oxidative stress while SEPP1, an extracellular protein, functions in selenium distribution [18,19]. Studies show that selenoproteins may play a critical role in reducing inflammation in adipose tissue [16,20]. Poor selenium status also impairs functional iron status as it contributes to the formation of methemoglobin, which contains ferric iron (Fe^3+^) and is unable to carry oxygen to tissues [21,22]. Studies also suggest that selenium is linked to anemia through the modulation of inflammation via the IL-6 pathway, the increased expression of heme-oxygenase 1 and oxidative stress [17,23,24,25]. Other researchers cite serum zinc as a potential mediator in this relationship [26]. 

Despite the increasing knowledge about the role of selenium in addressing inflammation, no study has investigated how selenium influences the chronic inflammation found in obesity and its relationship with hepcidin and iron status. The aim of this study was to compare selenium status, hepcidin concentration, and iron status biomarkers between individuals with normal weight and those with overweight/obesity, and to determine the associations among these biomarkers. We hypothesized that selenium status will be negatively correlated with hepcidin concentration and positively correlated with iron status biomarkers among study participants.

## 2. Materials and Methods 

### 2.1. Study Participants and Recruitment

**Figure 1:**
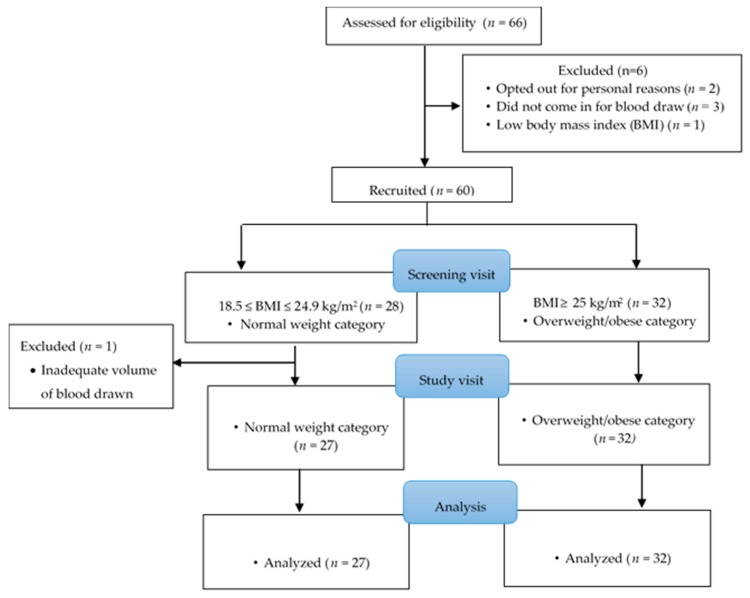
Subject recruitment.

Students and staff of the University of North Carolina, Greensboro (UNCG), USA, were recruited via mass emails in the summer of 2018. Out of a total of 129 subjects who responded to the mass emailing, 66 came for screening at the Cemala Foundation Human Nutrition Research Laboratories of the Nutrition Department (Figure 1). At the screening visit, potential subjects were provided with a copy of the informed consent form to read and sign if they agreed to participate in the study. The informed consent contained information on the study, as well as benefits and risks of participating. After the subjects had provided consent, they filled out the screening form with information on demographics, use of vitamin and mineral supplements, medical history, and any current medication. As part of the screening procedure, duplicates of their height and weight were also measured to estimate their body mass index (BMI) to assess their eligibility for the study. Subjects were eligible for the study if they were 18 to 49 years of age, with a BMI of 18.5 kg/m^2^ or over, with no history of inflammation-associated chronic diseases such as chronic kidney disease, cancer, heart disease, diabetes mellitus, and severe/mild hypertension. In addition, they were eligible if they were non-smokers, non-pregnant, non-lactating, and not taking any mineral/vitamin supplements or medicines that could interfere with iron and selenium status or inflammatory markers. Additionally, individuals who had donated blood within two months to the start of the study were excluded. 

We estimated that 58 subjects were needed to determine a significant association between selenium biomarkers and hepcidin concentration in a regression model assuming an R-squared of 0.5 (with a partial R-squared of 15% from selenium biomarkers) for a regression model with up to nine predictors at an alpha level of 0.05 and with a statistical power of 0.8. To meet this sample size, we recruited a total of 66 subjects with equal numbers of subjects having either normal weight or overweight/obesity. Out of this number, 2 subjects opted out for personal reasons, 3 of them did not come in for blood draw, 1 had a BMI < 18.5 kg/m^2^ at the screening visit, and 1 was excluded from the analysis because the required volume of blood could not be drawn at the study visit. Thus, 59 subjects were used in the final data analysis. The study was conducted in accordance with the Declaration of Helsinki, and the protocol was approved by the Ethics Committee of University of North Carolina at Greensboro (18-0173).

### 2.2. Study Design

The study was a cross-sectional study in which anthropometry, dietary intake data, and blood samples were obtained from subjects. Subjects who qualified based on the inclusion/exclusion criteria were provided with three-day dietary record forms and weighing scales to weigh and record foods eaten. They were to be completed and returned at the study visit. Instructions for completing the dietary record were provided by trained research assistants.

For the study visit, the participants were required to do a 10 h overnight fast. Dietary records were collected and height, weight, and other anthropometric measurements were taken. Approximately 30 mL of venous blood was collected to measure the concentrations of selenium biomarkers, C-reactive protein (CRP), and hematological parameters.

### 2.3. Anthropometry

A Seca 213 stadiometer (Seca, Hammer Steindamm, HH, Germany) was used to measure the standing height of the study participants following standard procedure, and weight in kilograms was measured using a Tanita BWB-800 weighing scale (Tanita Corporation of America, Inc., Arlington Heights, IL, USA) with subjects in light clothing [27].

### 2.4. Dietary and Nutrient Intake

To assess dietary intake using the three-day weighed dietary record, subjects were required to keep a log of all foods consumed on 3 non-consecutive days (2 weekdays and 1 weekend day) between the screening and study visits as previously described [28]. Dietary records were analyzed into nutrients and food groups using the Nutrition Data System for Research (NDSR software, version 2016, University of Minnesota, Minneapolis, MN, USA). To ensure reliability and accuracy of dietary records, a trained research assistant reviewed the dietary information with participants on the study visit and data were reviewed by another research assistant during data entry into NDSR. 

### 2.5. Sample Analyses

Fasting blood samples collected at the study visit were processed into serum, plasma, or whole blood for the measurement of selenium, iron status, and inflammatory markers as well as plasma hepcidin. Iron status biomarkers (ferritin, hemoglobin, total iron-binding capacity (TIBC), transferrin saturation, and serum iron) and CRP concentration were measured from serum or whole blood (in the case of hemoglobin) by LabCorp Laboratories (Burlington, NC, USA). With the exception of the serum and whole blood samples that were sent to LabCorp, all other samples were stored at the Department of Nutrition of UNCG at −80 °C and analyzed upon study completion. 

To measure plasma selenium concentration, plasma samples were digested with trace metal grade HNO_3_ overnight in a sand bath at 65 °C. The samples were then diluted to a final concentration of 2% v/v HNO_3_ using deionized water. The digested plasma samples were analyzed for plasma selenium concentration using an Agilent 8800 ICP-MS/MS from Agilent Technologies (Santa Clara, CA, USA) at the Department of Chemistry, Wake Forest University (Winston-Salem, NC, USA). Plasma concentrations of hepcidin, SEPP1, and GPX activity were measured using Enzyme Linked Immunosorbent Assay (ELISA) kits. Erythrocyte GPX activity was measured from red blood cell lysate obtained after the plasma was collected and the remaining red blood cells lysed. The GPX activity ELISA kit was obtained from Cayman Chemical (Ann Arbor, MI, USA), whereas the SEPP1 and hepcidin kits were obtained from MyBioSource (San Diego, CA, USA) and Peninsula Laboratories International (San Carlos, CA, USA) respectively. 

### 2.6. Statistical Analysis

Data were stratified according to subject BMI category (normal or overweight/obese). The primary outcome variables were the hematological parameters, whereas the independent variables were GPX activity and SEPP1. Means and standard deviations were reported for continuous variables with normal distributions. Median and interquartile ranges were reported for nutrient intakes. Percentages were reported for categorical variables. Ferritin, TIBC, SEPP1, plasma selenium, CRP, and hepcidin concentrations were log transformed to approximate normality. Student’s *t*-test was used to compare continuous variables between the two groups. The Wilcoxon rank-sum test was used to determine significant differences in nutrient intakes between the two subject groups. Linear regression analysis was used to determine the associations among selenium biomarkers, iron status, and hepcidin concentration adjusting for the potential confounders. Variables adjusted for in the regression analysis were age, gender, BMI category, ethnicity, and dietary selenium. Statistical significance was set at *p* ≤ 0.05. The R-software (Rx64 3.5.0.ink, R Foundation for Statistical Computing, Vienna, Austria) was used for data analysis.

## 3. Results

Out of the 59 participants included in the final analysis, a majority (71%; *n* = 42) were female, and 42% (*n* = 25) were Non-Hispanic Blacks. There were 46% (*n* = 27) of study participants with normal weight and 54% (*n* = 32) with overweight/obesity. About 19% (*n* = 11) of the participants had high C-reactive protein (CRP ≥ 3 mg/L). 

Table 1 shows the nutrient intake of participants. The median caloric intake was not significantly different between subjects with normal weight and those with overweight/obesity. Among the nutrients, vitamin C intake was lower in individuals with overweight/obesity (43.37 mg/day) compared to individuals with normal weight (88 mg/day), *p* = 0.028. All other nutrient intakes were not significantly different between the two groups (*p* > 0.05).

As expected, the mean BMI was significantly lower among individuals with normal weight compared with individuals with overweight/obesity, *p* < 0.001 (Table 2). The results showed a trend of high hepcidin and ferritin, and low selenium biomarkers, transferrin saturation, serum iron, and hemoglobin concentrations in the overweight/obese group although the differences were not statistically significant (*p* > 0.05). 

In multiple linear regression analysis, hepcidin concentration was significantly predicted by plasma GPX activity (β = −0.02, *p* < 0.01), SEPP1 concentration (β = −1.23, *p* = 0.028), and ferritin concentration (β = 1.01, *p* < 0.001), as shown in Table 3. Serum iron was predicted by ferritin concentration (β = 13.26, *p* = 0.035). Transferrin saturation was predicted by ferritin concentration (*p* < 0.001). Hemoglobin concentration was significantly predicted by ferritin concentration (*p* < 0.01). All the regression models were adjusted for age, gender, BMI category, and ethnicity. 

## 4. Discussion

In spite of the recognized association between selenium status and anemia, to our knowledge, no study has investigated the role of hepcidin in this relationship. The aim of this study was to investigate the associations among selenium status, hepcidin concentration, and iron status biomarkers among young adults with normal weight or overweight/obesity. 

Although a few studies have reported significantly higher concentrations of serum iron, transferrin saturation, and hemoglobin in obese adolescents compared to their non-obese counterparts (20,21), most studies have demonstrated that obesity is associated with iron deficiency, hypoferremia, and hyperferritinemia [29,30]. This is due to low-grade chronic inflammation which results in increased hepcidin concentration and concomitant iron sequestration [31,32]. There is supporting evidence that invading macrophages in obese adipose tissue sequester iron, resulting in iron overload [33]. While the results of this study showed the expected trends in serum iron, hemoglobin, transferrin saturation and ferritin, it is likely that the differences were not significant because there were no individuals with morbid obesity in the overweight/obese group, with most of them having overweight status. Studies comparing subjects with extreme obesity to those with normal weight may show statistically significant relationships among these variables. In addition, while higher hepcidin concentration is expected in obesity due to increased inflammation [34] and IL-6 concentration [35], the observed difference in this study between the normal weight and overweight/obese groups was not statically significant. 

Selenium is incorporated as selenocysteine into selenoproteins which exert immune and anti-inflammatory effects. Total plasma selenium is measured by the concentrations of the two predominant selenoproteins (GPX and SEPP1) in the body [36]. Low levels of selenium biomarkers are seen in obesity [37,38]. For example, there was a reduction in local SEPP1 expression in obese mice, and this is possibly because these selenoproteins, which regulate oxidative stress, are depleted due to the increased inflammation in obesity [39]. While some studies have reported a significant negative correlation between selenoproteins and BMI [23], our observed differences in plasma SEPP1 and GPX concentrations between the BMI categories were not significant, which may be because almost all subjects in our study were selenium replete. The daily median selenium intake for subjects in this study was 100.5 mcg compared to the recommended intake of 55 mcg for adults in the US [40]. Mean plasma selenium concentration of subjects was 113.1 ng/mL, also well above the reference level of 70 ng/mL [41]. Both plasma selenium concentration and dietary selenium intakes were not significantly different between the subjects with normal weight and those with overweight/obesity. 

Our key finding in this study was that hepcidin concentration was predicted by SEPP1 and plasma GPX activity in a regression model adjusted for potential confounders. The results from the model indicate that lower SEPP1 and plasma GPX activity were associated with higher hepcidin concentration. This observation supports one of the proposed mechanisms through which selenium deficiency increases anemia risk. This inverse relationship may be due to the fact that lower concentrations of selenoproteins are associated with increased oxidative stress, leading to inflammation. In inflammation, there is increased production of IL-6, which induces hepcidin production that leads to hypoferremia as described earlier, eventually resulting in anemia. Petrova et al. espoused this relationship and reported higher hepcidin and lower GPX concentrations in chronic kidney patients with ischemic stroke compared with healthy adults [42]. In these patients and among Alzheimer’s disease patients, hepcidin concentration was negatively correlated with superoxide dismutase and GPX [42,43]. To the best of our knowledge, this study is the first to discover this relationship among healthy individuals with normal weight or overweight/obesity. Other mechanisms by which selenium deficiency increases risk of anemia include increases in heme oxygenase 1, oxidative stress, and possibly through the effect of serum zinc as suggested by Houghton et al. [26].

It is interesting that, despite observing this relationship between selenoprotein concentrations and hepcidin, we did not find a significant association between selenoproteins and iron status biomarkers, as shown by other studies [22,23]. Since this relationship is well documented, our lack of significant observations may be because the study was not powered to determine those associations as significant. The sample size was calculated to determine a 15% variability in hepcidin concentration attributed to selenoproteins which is close to what we observed (16%). Other possible reasons for the non-significant association between circulating iron and selenium biomarkers may be because few subjects (17%; *n* = 10) in this study had anemia, with 7 of these subjects in the overweight/obese category. Moreover, none of these anemia cases were due to inflammation. Studies show that pathological conditions of induced anemia in mice is linked to reduced glutathione peroxidase and selenoprotein W concentrations, suggesting that these associations may be more evident in anemia [24]. 

## 5. Limitations

Causal inference cannot be drawn from this study because it is a cross-sectional study. Additionally, other parameters that can influence the antioxidant and hepcidin levels of subjects were not measured in this study. Most of our participants were selenium sufficient and none had anemia of inflammation, hence the inability to observe obvious trends in selenium and iron status markers as statistically significant. In this study, GPX activity in erythrocytes was measured to determine intracellular GPX pools and long-term selenium status. However, erythrocytes are collected with blood and, if not treated properly, may be less advantageous over plasma as a specimen for assessing selenium status. Also of note is the likely interaction of glutathione with heme iron impacting the results [41]. To mitigate this shortcoming, GPX activity in plasma was also measured. 

## 6. Conclusions

This study is novel in showing an inverse association between hepcidin and selenium status among healthy subjects with normal weight or overweight/obesity. Additional studies on this relationship, including individuals with morbid obesity (who due to adipose tissue inflammation are prone to selenium deficiency and impaired iron status), are needed. Furthermore, using animal models of obesity will be helpful in investigating mechanistic relationships among selenium status, hepcidin, and iron status. Studies are also needed to investigate whether improving selenium status among individuals with selenium deficiency will improve iron status via hepcidin. 

## Figures and Tables

**Table 1:** Daily nutrient intakes among young adults with normal weight or overweight/obesity ^1^.

Nutrient/Calories	Normal (*n* = 27)	Overweight/Obese (*n* = 32)	Total	*p*-Value ^2^
Caloric intake, kcal	1844 (1423, 2611)	1878 (1347, 2234)	1875 (1357, 2304)	0.667
Total fat, g	66.28 (56, 107)	80.67 (53, 98)	72.01 (54, 104)	0.982
Protein, g	70.52 (50, 97)	73.1 (52, 96)	73.16 (51, 97)	0.994
Selenium, mcg	99.42 (70, 151)	102 (83, 153)	101.82 (79, 152)	0.645
Iron, mg	17.24 (10, 20)	12.41 (10, 16)	13.33 (10, 19)	0.272
Zinc, mg	8.97 (6, 16)	9.28 (7, 12)	9.19 (6, 14)	0.886
Vitamin A, mcg	352.51 (214, 442)	318.71 (180, 427)	352.51 (190, 430)	0.667
Vitamin C, mg	88 (52, 124)	43.37 (19, 95)	57.44 (34, 103)	0.028 *
Vitamin E, mg	11.22 (7, 21)	9.39 (7, 12)	10.53 (7, 13)	0.19

* *p* < 0.05; ^1^ Values are median (25th, 75th percentile); ^2^ *p*-values are based on the Wilcoxon rank-sum test.

**Table 2:** BMI and biochemical markers among young adults with normal weight or overweight/obesity ^1^.

BMI/Biochemical Markers	Normal (*n* = 27)	Overweight/Obese (*n* = 32)	Total	*p*-Value ^2^
	Mean	Mean	Mean	
BMI, kg/m^2^	22.43 ± 0.34	29.28 ± 0.74	26.15 ± 0.62	<0.001 ***
Erythrocyte GPX activity, nmol/min/mL	1001 ± 76	1077 ± 75	1042 ± 523	0.766
Plasma GPX activity, nmol/min/mL	84.29 ± 3.59	79.48 ± 3.31	81.75 ± 2.41	0.169
Hemoglobin ^3^, g/dL	13.48 ± 0.30	13.25 ± 0.35	13.35 ± 0.23	0.616
Transferrin saturation, %	29.33 ± 2.98	26.59 ± 2.21	27.85 ± 1.79	0.456
Serum iron, µg/dL	98.37 ± 8.96	91.06 ± 7.55	94.41 ± 5.71	0.528
Ferritin ^4^, ng/mL	36.2 (13, 101)	41.07 (13,136)	38.76 (13, 118)	0.677
TIBC ^4^, µg/dL	350.37 (284, 432)	347.56 (296, 407)	348.84 (287, 420)	0.874
Plasma selenium ^4^, ng/mL	114.38 (90, 145)	112.06 (92, 137)	113.1 (91, 141)	0.363
SEPP1 ^4^, ng/mL	360.77 (290, 450)	352.13 (276, 446)	356.05 (284, 450)	0.347
Hepcidin ^4^, ng/mL	9.95 (3, 29)	15.09 (3, 67)	12.47 (3, 47)	0.218

BMI, Body mass index; TIBC, Total iron-binding capacity; SEPP1, selenoprotein P. *** *p* < 0.001; ^1^ Values are mean ± SD; ^2^ Missing data (*n* = 1); ^3^ *p*-values are based on independent *t*-test; ^4^ Values are geometric means (± 1SD).

**Table 3:** Association between iron status biomarkers and selenoproteins in young adults with normal weight or overweight/obesity.

Demographic and Biochemical Indicators	Hepcidin ng/mL (*n* = 55) ^1^		Serum Iron, ug/dL (*n* = 55)		Transferrin Saturation, % (*n* = 55)		Hemoglobin, g/dL (*n* = 54)	
Predictors	β ± SE	*p*	β ± SE	*p*	β *±* SE	*p*	β ± SE	*p*
Plasma GPX activity, nmol/min/mL	−0.02 ± 0.01	0.009	−0.24 ± 0.33	0.474	−0.03 ± 0.09	0.705	−0.01 ± 0.01	0.208
Ferritin, ng/mL ^1^	1.01 ± 0.12	<0.001	13.26 ± 6.07	0.035	6.58 ± 1.59	<0.001	0.51 ± 0.16	0.002
C-reactive protein, mg/L ^1^	0.02 ± 0.09	0.849	−6.47 ± 4.53	0.161	−2.23 ± 1.18	0.067	−0.08 ± 0.12	0.483
Erythrocyte GPX activity, nmol/min/mL	−0.00 ± 0.00	0.776	−0.00 ± 0.01	1.000	0.00 ± 0.00	0.839	0.00 ± 0.00	0.808
Selenoprotein P, ng/mL ^1^	−1.23 ± 0.54	0.028	−26.15 ± 26.74	0.334	−4.26 ± 6.99	0.545	−0.93 ± 0.70	0.192
Gender								
Male	−0.38 ± 0.32	0.245	20.92 ± 15.80	0.193	6.07 ± 4.13	0.149	1.94 ± 0.41	<0.001
Age, years	0.01 ± 0.04	0.887	1.65 ± 1.85	0.376	0.37 ± 0.48	0.454	0.05 ± 0.05	0.343
Weight status, kg/m ^2^								
Overweight/obese	0.23 ± 0.26	0.386	0.14 ± 12.77	0.991	−0.07 ± 3.34	0.984	0.30 ± 0.34	0.383
Ethnicity								
Black	−0.12 ± 0.31	0.699	−10.00 ± 15.17	0.513	−3.92 ± 3.96	0.328	−1.27 ± 0.41	0.003
Hispanic	−0.46 ± 0.40	0.262	−25.83 ± 19.88	0.201	−9.34 ± 5.19	0.079	−0.73 ± 0.53	0.173
Other Race ^2^	0.33 ± 0.37	0.376	−4.22 ± 18.22	0.818	−4.93 ± 4.76	0.307	−0.54 ± 0.49	0.273

^1^ Variables were log transformed before analysis; ^2^ Other Race are Asians, Arabs, Multiracial, and Persians.
